# Accelerating compartmental modeling on a graphical processing unit

**DOI:** 10.3389/fninf.2013.00004

**Published:** 2013-03-18

**Authors:** Roy Ben-Shalom, Gilad Liberman, Alon Korngreen

**Affiliations:** ^1^The Leslie and Susan Gonda Interdisciplinary Brain Research Center, Bar-Ilan UniversityRamat Gan, Israel; ^2^The Mina and Everard Goodman Faculty of Life Sciences, Bar-Ilan UniversityRamat Gan, Israel

**Keywords:** CUDA, GPU, NEURON, ILP, parallel computing, compartmental modeling

## Abstract

Compartmental modeling is a widely used tool in neurophysiology but the detail and scope of such models is frequently limited by lack of computational resources. Here we implement compartmental modeling on low cost Graphical Processing Units (GPUs), which significantly increases simulation speed compared to NEURON. Testing two methods for solving the current diffusion equation system revealed which method is more useful for specific neuron morphologies. Regions of applicability were investigated using a range of simulations from a single membrane potential trace simulated in a simple fork morphology to multiple traces on multiple realistic cells. A runtime peak 150-fold faster than the CPU was achieved. This application can be used for statistical analysis and data fitting optimizations of compartmental models and may be used for simultaneously simulating large populations of neurons. Since GPUs are forging ahead and proving to be more cost-effective than CPUs, this may significantly decrease the cost of computation power and open new computational possibilities for laboratories with limited budgets.

## Introduction

Rall (1964) introduced compartmental modeling of neurons, pioneering the use of digital computers in neuronal simulations. Two decades later Hines developed an efficient method for solving the differential equation systems underlying compartmental models (Hines, 1984). Since then many research groups have developed software packages using Hines' theory (De Schutter, 1989; Hines, 1989; Wilson et al., 1989; Manor et al., 1991), but today GENESIS (Wilson et al., 1989) and NEURON (Carnevale and Hines, 2006) are the two most commonly used.

A multi-compartmental model demands considerable computation resources, especially where multiple instances of the model are used for statistical analysis or optimization algorithms (Keren et al., 2005, 2009; Huys et al., 2006; Van Geit et al., 2008). Intensive computation results in long runtime and forces compromises in model complexity or data size. A common means of reducing simulation runtime is to disperse the computation onto different cores by using computer clusters (Keren et al., 2005, 2009; Druckmann et al., 2007; Van Geit et al., 2008). Hines et al. (2008) added the functionality of splitting a single neuron model into sub-trees and sending each to a different processor, achieving a linear speed related to the number of processors used. This method is designed for CPU clusters or supercomputers such as the Blue Gene/L (Gara et al., 2005) which are very costly.

Recent advances in the field of graphical processing units (GPUs) have brought parallel computation to end-users with higher cost-effectiveness than with CPUs (Owens et al., 2007; Nickolls et al., 2008; Ryoo et al., 2008). GPUs have been utilized for highly demanding applications in hydrodynamics (Harada et al., 2007), molecular dynamics (Harada et al., 2007), astrophysics (Nyland et al., 2007), and many other fields (Owens et al., 2007, 2008). In neuroscience GPUs have been used to simulate networks of spiking neurons (Fidjeland et al., 2009; Nageswaran et al., 2009) and processing of data images (Jeong et al., 2010). We have previously used GPUs to accelerate simulations of ion channels for data fitting and have achieved a speed up to a thousand times faster than a single CPU (Ben-Shalom et al., 2012). Here we present a parallel algorithm for simulation of multi-compartmental models of neurons on GPUs and validate its correctness and high speed gain compared to NEURON on various neuron topologies and membrane models.

## Methods

### Simulation environment

All simulations were performed on a PC containing an AMD Phenom^(TM)^II X4 920 processor (2.81 GHz with 8 MB) running Windows 7. GPU simulations were performed on NVIDIA's Tesla C2075 (NVIDIA, 2009) with 14 multiprocessors (448 CUDA cores) and 6 GB of GPU memory. Code was written on Visual Studio using CUDA 4.2, the program was debugged and profiled with NSIGHT (NVIDIA, 2011a). The source code is freely available from the authors under the GNU public license—http://code.google.com/p/neurogpu.

Since NEURON is the most mature and widespread simulation environment for neurophysiological simulations, we implemented our application as closely as possible to NEURON with adjustments required for GPU coding. We validated the results against NEURON 7.2 (Carnevale and Hines, 2006). We set NEURON to work in the Backward Euler scheme that we implemented using CUDA. NEURON procedures were written using the topology and fmatrix functions (Carnevale and Hines, 2006) for exporting NEURON data to readable files. Data files were read by the application and the equation system was built based on this data. Parameters describing the membrane mechanisms such as Nernst potential, model parameters, stimulus parameters, etc., were also exported to the application from NEURON.

The simulation protocol used here was a current-clamp protocol with time step sizes of *dt* = 0.1 ms and *dt* = 0.01 ms for a period of *t* = 10 s, injecting current of −1 nA for passive stimulation and 1 nA for stimulation that provoked an action potential. In simulation where there were more than two stimuli the current started at −1 nA and increased in each sweep by 0.3 nA (see Figure 5A). We used two membrane models: a passive membrane (Carnevale and Hines, 2006) and the Hodgkin–Huxley model (Hodgkin and Huxley, 1952; Carnevale and Hines, 2006). The passive membrane model solves one equation:

(1) I=g(v-e)

where *I* is the current passing through the membrane *g* is the membrane conductance *v* is the membrane voltage and *e* is the Nernst potential. In terms of computation time, this is almost equivalent to not using a model at all. On the other hand, the Hodgkin–Huxley model solves the gate differential equations requiring more computational effort (Figure 3; see Carnevale and Hines, 2006). With the passive membrane model, the conductance was 0.001 S/cm^2^ along the whole neuron. With the Hodgkin–Huxley model the conductances at the soma were Na^+^ −24 S/cm^2^ and K^+^ −2.88 S/cm^2^. At the dendrites Na^+^ conductance was 0.12 S/cm^2^ and K^+^ conductance was −0.036 S/cm^2^. To allow realistic comparison, table look up optimization were turned-off in NEURON by setting usetable_hh = 0. Four topologies were used: two reconstructed layer 5 pyramidal neurons from the rat cortex adopted from previous studies (Keren et al., 2005, 2009); one fork topology (Figure 4A) and several binary trees (Figure 4B) which were artificially programmed in NEURON. Binary trees were built with depths from 5 to 10, where each node had one segment, giving 2^depth^ elements in the matrix (equation system).

### CUDA implementation

Kernels are executed in thread warps (32 threads) on the GPU multiprocessors, where warps are the true parallel unit of the device. When other elements, such as several blocks, multi-threading or several multiprocessors work in parallel, they resemble multi-core CPU, rather than a parallel SIMD (single instruction multiple data) device. Each thread has a unique 3D address within a block and multiple blocks are ordered in a grid. The dimensions of the grids and blocks are specified when calling a kernel (NVIDIA, 2011b). Grids and blocks are used for organizing the threads in a logical hierarchy for execution at the GPU device.

Here a neuron was simulated by a thread warp, with each segment computed by a single thread in the warp. If the neuron had more than 32 segments, each thread was assigned to multiple segments using instruction level parallelism (ILP). Hence, a simulation of a current-clamp trace (stimulating the neuron once) was dispersed to the 32 threads of a warp. Multiple membrane potential traces were organized in a block; the size of the y-axis of a block was determined by the number of traces in the protocol. Finally, multiple neurons, each represented as a block, were ordered in a grid.

#### Global and shared memory organization

We used the global, shared, and local (registers) memories of the GPU. Global memory is slow and large, its scope being the application, and all blocks have access to it. In our application the global memory stored the membrane potential response of the neuron and functioned as bridge to transfer memory from the CPU to the shared memory. Shared memory is limited in size, specifically on the Tesla C2075 to 48 kilobytes but is very fast and its scope is the block—threads in the same block can access the same shared memory. We divided the shared memory into two sections: framework and per-stimulus. The framework section was used by all warps in the block and included information related to the neuron's topology. This section was not subject to change during the simulation. The per-stimulus section held additional neuronal parameters, as well as buffer memory used for the matrix calculation, such as the right and left hand sides of the equation system (Hines, 1984). Each trace in the protocol, hence each warp, had an independent stimulus section within the shared memory. The shared memory was further used for passing the resultant voltage of each trace from registers to the global memory via a buffer array of 32 (Warp size) elements. Every 32 time steps each thread in the warp transferred the voltage of one time step to the global memory in a coalesced manner (NVIDIA, 2011b). Local memory was used for local variables, including the states of the membrane mechanisms and for using ILP.

#### Instruction level parallelism (ILP)

One of the main challenges of programming in CUDA is masking memory transfers' latencies from the slow global memory. In order to mask memory latencies, transfers should occur while the multiprocessor executes math operations on the available memory. Volkov (2010) suggested that ILP aids reaching the theoretical computational peak of the GPU. In ILP code each thread executes instructions for multiple memory elements as opposed to non-ILP code where each thread deals with a single memory element. When each thread is accessing multiple memory elements the advantage is double: first each memory transfer is larger and there are less memory transfers, second the multiprocessor has more available memory for math operations within each thread. Thus, when using ILP, memory latencies are better masked by math operations. We used ILP for simulating one membrane potential trace in the neuron. When the neuron had only 32 segments each thread in the warp simulated one segment. However, when the neuron was larger, each thread simulated more segments in an ILP manner. Each thread had an address within the warp between 0 and 31, the thread calculated all the segments in the neuron so that their remnant of 32 was equal to its address (e.g., if the neuron had 96 segments each thread calculated 3 segments, such that for example, a warp addressed 3 would calculate the segments indexed 4, 36, and 68).

## Theory and results

Simulating a compartmental model for a neuron may be divided into calculating the membrane mechanisms for each segment and solving the equation system which describes the current diffusion. While the first step falls under the category of “embarrassingly parallel,” the second step requires solving a *quasi* tri-diagonal matrix which is difficult to solve in parallel. For an unbranched neuron, the problem reduces to solving a (pure) tri-diagonal equation system. This problem has wide application and has received much attention from the mathematics and engineering communities (Stone, 1973; Wang, 1981; Hegland, 1991). We have focused on a rather early suggestion by Stone (1973) which, while not presenting the complexity benefits of more recent research, has the advantage of being simple and flexible enough to accommodate modification for the back-substitution phase even in a branched cable problem (resulting in a *quasi* tri-diagonal matrix). Another advantage is the low overhead; speedup is evident even when using a small number of processors (warpsize in GPUs consists of 32 threads). Several parallel solutions for the branched cable problem have been suggested (Mascagni, 1991; Migliore et al., 2006; Plesser et al., 2007; Hines et al., 2008). These solutions are for a multi-kernel CPU, a small grid or a supercomputer such as the IBM BlueGene/L (Markram, 2006) and are not tailored for the GPU SIMD architecture hence they could not fully utilize the advantages of GPUs.

The core of the parallelization problem lies in the parallel solutions for the equation system *Hx* = *b*, as described by Hines (1984). Hines also describes how a neuron can be represented by an almost tri-diagonal matrix. We refer to the almost tri-diagonal matrix resulting from a branched neuron as a *quasi tri-diagonal* matrix. The matrix has elements of the three diagonals where branching occurs. Stone (1973) introduced a parallel solution for the tri-diagonal matrix, which in our case represents an unbranched neuron. The tri-diagonal matrix is solved in three stages: (a) *LU* decomposition *H* = *LU*, where *L* and *U* are bi-diagonal matrices; (b) solution of *Ly* = *b*; (c) solution of *Ux* = *y*. Hines (1984) introduced a serial algorithm solving both a and b stages by applying a bottom-up scan of the equation system (which he called triangularization), while a top-down scan solved the third stage [referred to as back-substitution in Hines (1984)]. Note that for a *quasi* tri-diagonal matrix the related *L* and *U* matrices are not bi-diagonal.

We show that solving the back-substitution stage (c) can still be done in parallel using a simple modification of Stone's algorithm to this step, while branching points naturally divide the problem. In both the triangularization and back-substitution steps, the computation for different branches is independent, given that they do not precede each other in the tree structure. We use this feature for parallelizing the triangularization step. For the back-substitution step, we suggest two options. The first option is based on the independence between branches (which we call branch-based parallelization). The second option is based on our modification of Stone's method (which we call segment-based parallelization). The branched-based parallelization is very similar to what Hines (1994) suggested to use for multiple data stream machine as the CRAY-YMP.

### Branch-based parallelization

**Figure 1:**
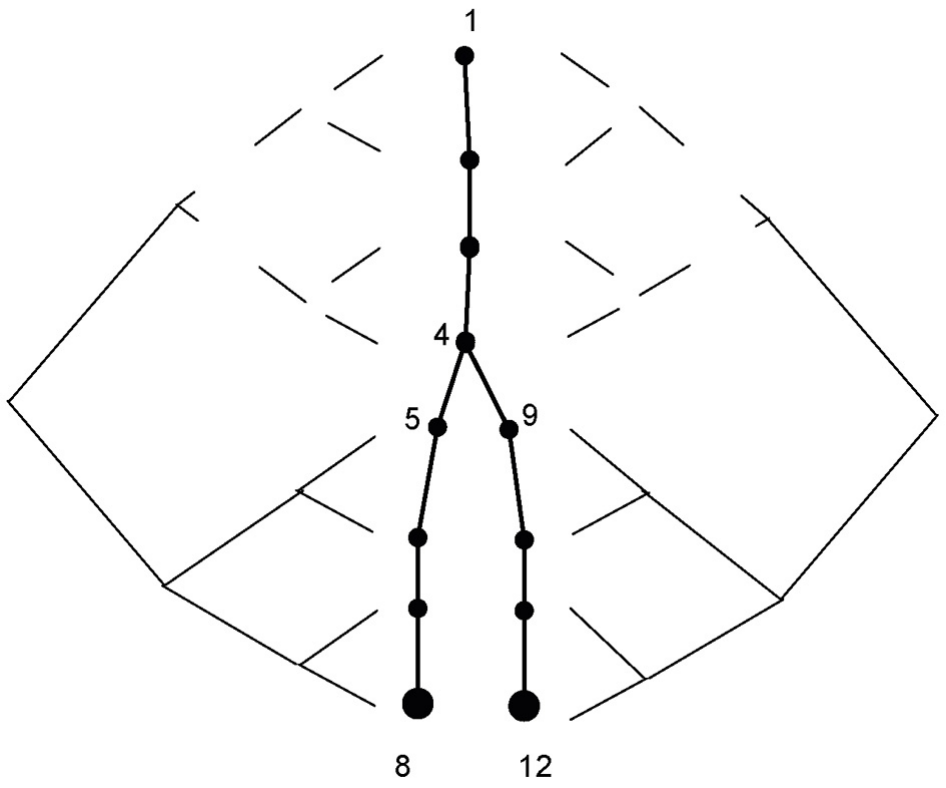
**Segment-based parallelism.** The calculation for node 8 involves only segments 1–8, and is carried out in parallel according to Stone's algorithm. The calculation for node 12 involves segments 1,2,3,4,9,10,11,12 and is of the same nature as the calculation for node 8, but with different indexing. Calculations for segments 1–4, which are marked by the dashed line, are the same on both sides and are carried out only once.

A neuron can be described as a tree, and we assign a *level* for each branch according to its distance from the root (e.g., soma = 1, see Figure 1 where the soma compartment has 4 segments). After indexing the branches in this way, we can independently apply both triangularization and back-substitution for all branches at the same level of the tree. All branches in a level can be simultaneously solved, if the branches in the preceding level have already been calculated (i.e., levels that are higher than the current level in the triangularization stage, and levels that are lower than the current level in the back-substitution stage). With this method, the levels are calculated serially but the branches in the level are calculated simultaneously. The triangularization stage requires additional computation; each branching in the tree incorporates the information from all of a node's child nodes. This can be done simultaneously by assigning different processers to handle each branch in a particular level. Finally, the order of complexity for simulating one time step is:

(2) $$\begin{array}{l} O(dt)=\sum_{\text{level }=\;1}^{\text{depth}}\underset{\text{branch }\in \text{ level}}{\max}\left(L\left(\text{branch}\right)\right)\\ \;+\sum_{\text{level }=\;2}^{\text{depth}}\underset{\text{branch }\in \text{ level}}{\max}\left(D\left(\text{branch}\right)\right). \end{array}$$

where *dt* is the time step, *L* is the number of segments in the branch, and *D* is the degree of the branch (number of children).

We expect that this approach would perform better for neuron that has many branches with similar number of segment among the branches of the same level.

### Segment-based parallelization

When this method is used the triangularization stage is the same as described above. The difference is at the back-substitution stage of the algorithm. The node's value solely depends on its parent's value. For an unbranched cable, the parent's index is the consecutive index. Stone's algorithm solves the equations in log*N* parallel steps, where processor *i* after *j* steps has incorporated the values from the 2^*j*^ nodes preceding to *i* (as described at Figure 1). Thus, after log*N* steps each processor has integrated the values from all relevant nodes. A similar process is implemented for the branched cable back-substitution: the problem properties remain the same except for the unique path from node *i* to its root, which may include non-consecutive indices [the indexing algorithm for a branched cable is described at Hines (1984)]. Finally all nodes have the correct index of node/processor which contains the relevant value for the *j*^th^ step of the algorithm is calculated before the run of the simulation (in an iterative manner, finding the 2^*j*−1^ ancestor of *i* on the unique path) and stored in the device's shared memory. For *N* segments, the complexity is therefore:

(3) $$O(dt)=\frac{N}{W}\log_{2}m$$

where *N* is the number of segments, *m* is the maximal root-leaf distance and *W* is the warpsize. (The 1/*W* factor was left inside to describe how the architecture, i.e., number of warps per multiprocessor, affects the runtime).

Both branch-based and segment-based parallelization approaches are tailored to the GPUs architecture. Both approaches maintain controlled use of shared memory and simultaneously compute single instructions on multiple data (SIMD). The actual computation time on the GPUs may vary from the theoretical complexity due to the special architecture properties and optimizations done by the compiler. Computation time for various instances of the problem will be analyzed in the following subsections.

## Correctness/error analysis

**Figure 2:**
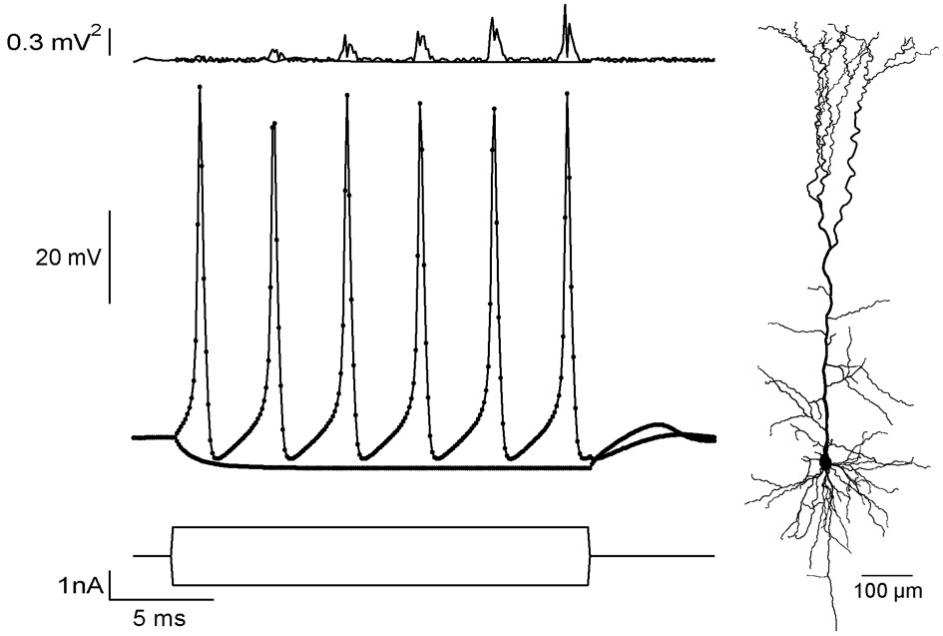
**Comparing current-clamp simulations in NEURON and CUDA.Bottom**: Current-clamp protocols for passive and active responses. **Middle**: Voltage responses of a pyramidal neuron simulation (morphology at the right) using NEURON (dots) and CUDA (solid line). **Top**: Squared error values between NEURON and CUDA platforms for both passive and train simulations.

To validate correctness of the CUDA application, we simulated a current-clamp protocol using NEURON (Carnevale and Hines, 2006) and exported it to CUDA. We used the reconstructed morphology of a pyramidal neuron from a previous study (Keren et al., 2009) (Figure 2, right) with a Hodgkin–Huxley membrane mechanism (Hodgkin and Huxley, 1952; Carnevale and Hines, 2006). Two stimulations were applied (Figure 2, lower traces), one to produce a passive response (stimulus amplitude was −1 nA) and one for producing an action potential train (stimulus amplitude was 1nA). Figure 2 shows the voltage responses of both simulations (middle), along with error curves (top). Our application showed excellent agreement with NEURON. For the 1 nA sweep, the maximal and average root-mean-square errors were 0.80 mV^2^ and 0.20 mV^2^, respectively, when using the long-time step (*dt* = 0.1 ms). With a shorter-time step (*dt* = 0.01 ms) maximal and average root-mean-square errors were −0.22 mV^2^, 0.03 mV^2^, respectively.

The apparent increase in the error (Figure 1, up) during the spike train is due to a small linear shift in time, calculated to be 0.4 μs per spike, that is due to the use of single-precision arithmetic. Both time step value gave negligible disagreement for the passive stimulation (Figure 2, top). This validation of the application against NEURON assured the correctness of our method.

### Model workload

**Figure 3:**
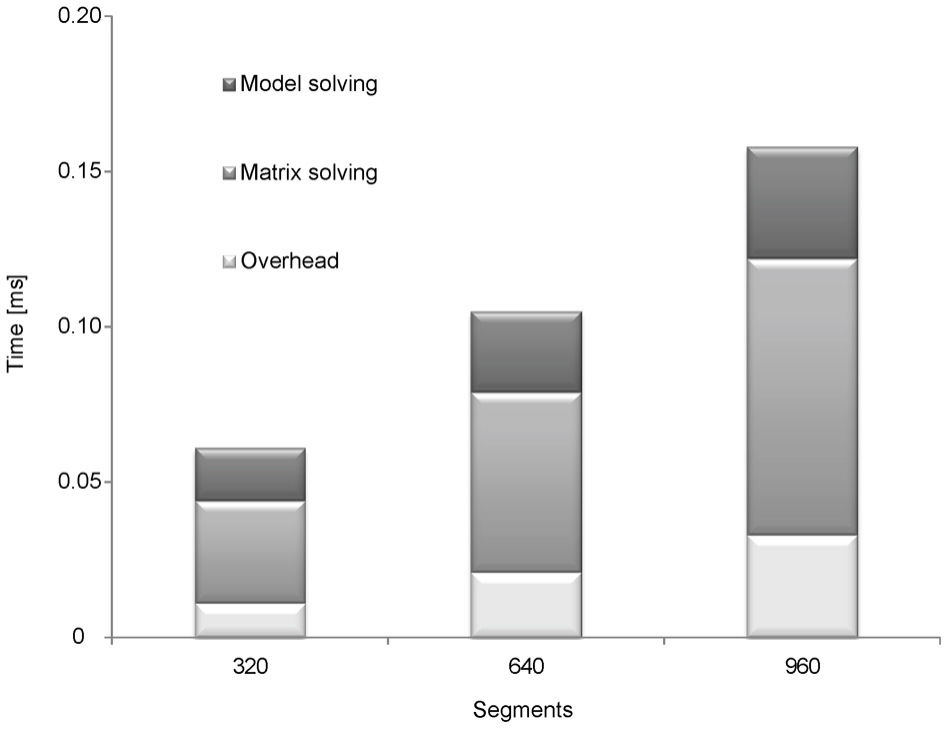
**Analyzing the runtime of matrix and model solving.** Four different simulations were used to analyze the runtime of model solving and solving the equation system (matrix): 1, full simulation; 2, simulation with a passive model; 3, simulation without solving the matrix (Hodgkin–Huxley model); 4, simulation with a passive model and without solving the matrix. To calculate the runtime of matrix solving the runtime of simulation with a passive model was subtracted from the full simulation. To calculate the runtime of model solving the runtime of simulation without matrix solving was subtracted from full simulation. The simulations were ran on fork morphologies with 320, 640, and 960 segments.

**Figure 4:**
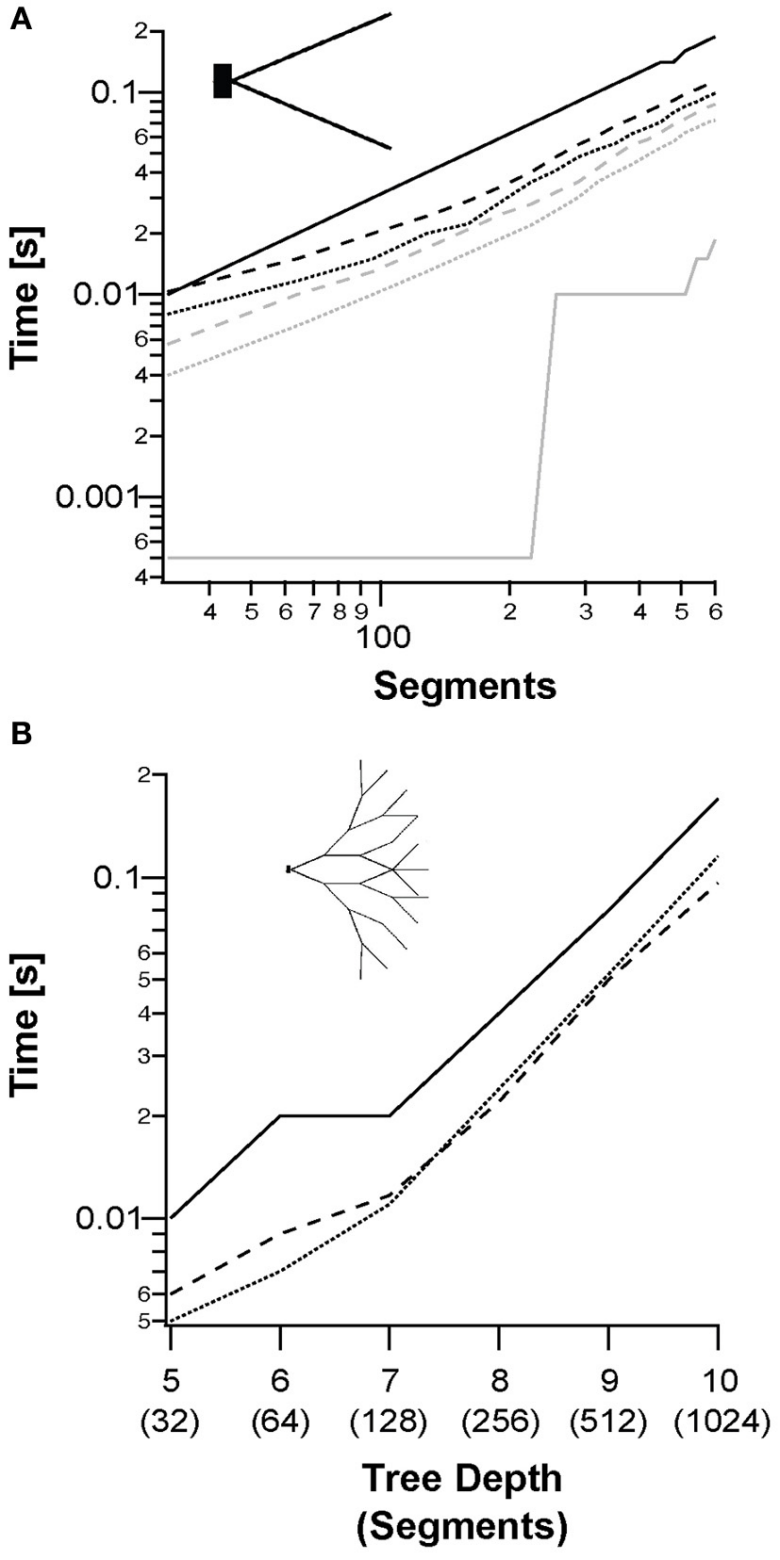
**Simulation of a single sweep of a single neuron of different topologies, models, and scale. (A)** Median runtimes of a fork morphology (inset) with increasing number of segments for: NEURON simulation (solid lines); segment-based parallelization (dotted line); and branching-based parallelization (dashed line). The Hodgkin–Huxley model is shown in black and the passive model in gray. **(B)** Median runtimes of full binary tree morphologies (inset) using the Hodgkin–Huxley model with increasing tree depth. The passive model is not shown for clarity.

Each time step the application solves the membrane mechanism models and adds their conductance and current contributions to the current equation system (Hines and Carnevale, 1997, 2000). When all mechanisms are solved, the application can update the equation system and solve it. The result from the equation system is the membrane voltage of each segment of the neuron at the current time step. The main computation effort for advancing a time step in the simulation derives mostly from three operations: calculating the membrane mechanism, setting the equation system and solving it. We analyzed the model workload in two steps. First, we analyzed the runtime for solving the matrix (which represents the equation system) and the model (Figure 3). Second, we compared the runtimes of full simulations using different models on NEURON and CUDA (Figure 4).

The runtime of solving the matrix was analyzed by eliminating the relevant part of the code and running the simulation without it. Runtime of solving the model was analyzed using the passive model, which hardly requires any computational effort. We conducted another run using the passive model but without solving the matrix to estimate the runtime for the rest of the code (Figure 3). Next, we subtracted the runtime of the simulation without the code for solving the matrix from the runtime of a full simulation to calculate the runtime for solving the matrix. Similarly, we subtracted the runtime of the simulation with the passive model from the simulation of the Hodgkin–Huxley model to calculate the runtime of computing the model.

These tests were repeated with fork morphology of 320, 640, and 960 segments (the morphology is displayed in Figure 4A). The results are shown in Figure 3. Most of the runtime was used for solving the matrix (54.1% at 320 segments 55.2% at 640 segments and 56.3% at 960 segments). With an increasing number of segments (heavier simulations) solving the matrix took a more significant share of the total runtime. This result agrees with the theoretical analysis; as the per-segment model calculation and updating the equation system run in linear time, the complexity of solving the matrix is super-linear.

The runtimes of whole simulations were compared with NEURON, using an increasing number of segments. Combinations of the back-substitution method with the different models and topologies are compared in Figure 4. Figure 4A shows the simulation runtime for a single trace as a function of the number of segments in the neuron. We checked runtimes of both segment-based and branching-based parallelization approaches using both the passive and Hodgkin–Huxley models. These were compared to NEURON runtimes. NEURON outperformed both parallelization methods with the passive model, (slope ratio of 5.3:1). However, for the Hodgkin–Huxley model, the GPU was faster (slope ratio of 1:1.8). For the fork topology, the segment-based parallelization performed best (Figure 4A). Figure 4B shows the runtimes for Hodgkin–Huxley simulation on a binary tree topology. In this topology, with small depths of less than 7, the segment-based parallelization still performed better than the branching-based parallelization. With depths greater than 7 there was enough branching in the tree for branching-based parallelization to outperform the other simulations.

### Simulating multiple traces

**Figure 5:**
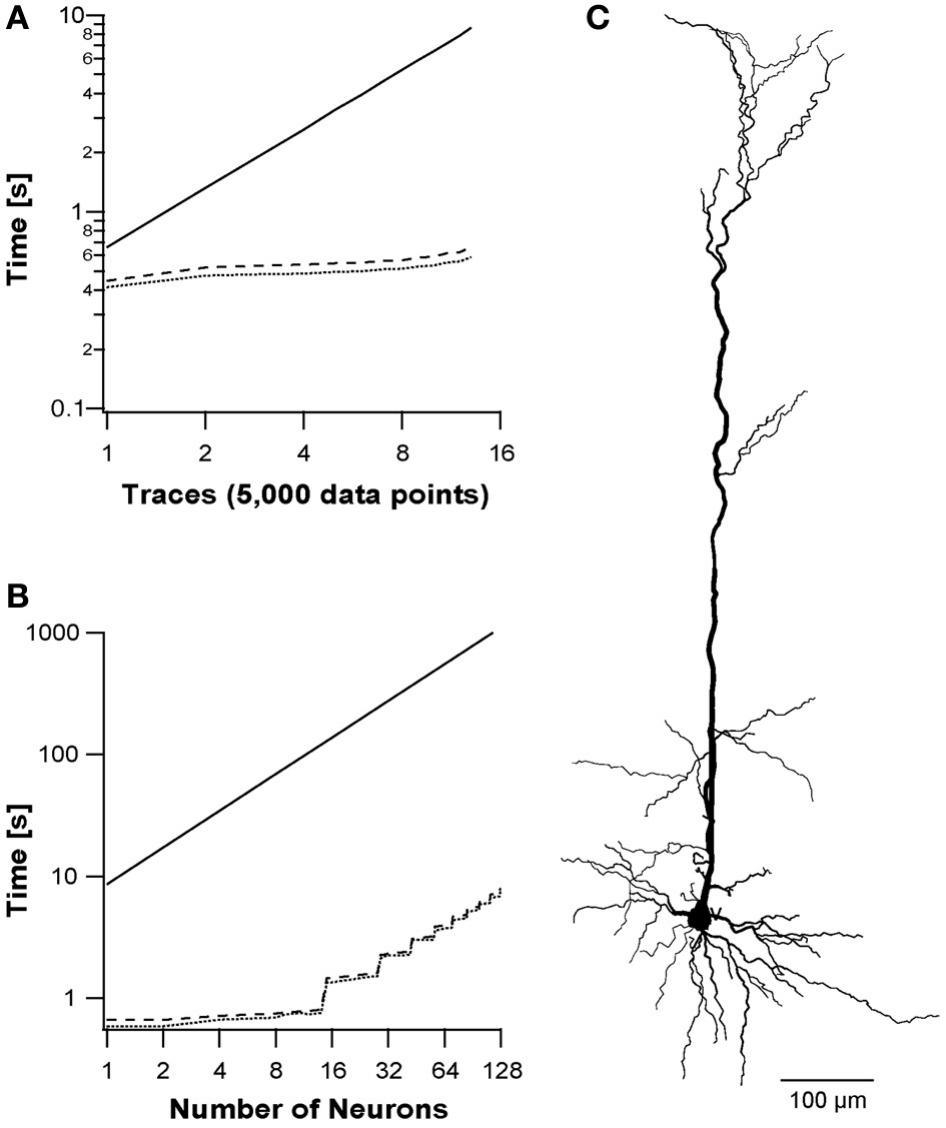
**Simulation of multiple sweep protocol and multiple neurons.** A simulation protocol using reconstructed pyramidal neuron morphology with 112 branches **(C)** and a varying number of sweeps, where each sweep included 5000 data points. **(A)** Run times of increasing number of sweeps using three simulations: NEURON simulation (solid line); segment-based parallelization (dotted line); branching-based parallelization (dashed line). The y-axis shows the runtime on a log scale. **(B)** Run times of the three simulations on multiple neurons, where each neuron consists 13 traces (65,000 data points) as described in **(A)**.

Current-clamp experiments usually use multiple traces to evaluate the reaction of the neuron to different physiological conditions. As described in the Methods section, each thread block was organized such that the CUDA block's y-index described a different trace. The runtime analysis for simulation of multiple traces in a single multiprocessor is presented in Figure 5. We used current-clamp protocols, where each trace included 5000 data points, and stimulated the neuron with increasing number of traces from 1 to 13. The different back-substitution approaches were used with the different protocols and compared to NEURON simulations. While NEURON's runtime is a simple factor of the number of traces (Figure 5A), the CUDA runtimes for up to 13 traces were sub-linear, as the runtime for 13 traces was only 50% slower than the runtime for a single trace. GPUs' runtimes increase sub-linearly due to optimization of multiprocessor context switching, leading to a performance factor of 14.6× on the tested morphology with the Hodgkin–Huxley model compared to NEURON.

### Multiple neuron simulations

Finally, we utilized all 14 multiprocessors by simulating several neurons on different multiprocessors using the grid of blocks (NVIDIA, 2011b). We stimulated each neuron with 13 traces (65,000 data points) resulting in a block of 32 × 13 threads. Different number of neurons were ran and the performance of the two back-substitution approaches were compared with NEURON. NEURON's runtime was extrapolated from 3 points since they were linear with the number of neurons. We simulated 1–128 neurons. Above 128 neurons the GPU simulation runtimes increased linearly with neuron number, while below 14 neurons GPU runtimes remained virtually equal (Figure 5B). Above 14 neurons, the runtime depended on the longest running multiprocessor, i.e., the number of 14-neuron units. The method achieved a 150-fold performance factor for a realistic configuration of morphology and stimulation protocol with several neurons.

## Discussion

We have developed a parallel algorithm for simulating multi-compartmental models of single neurons on GPU devices. Simulations using our application were accurate and achieved a peak running speed 150 times faster than a single CPU running NEURON (Carnevale and Hines, 2006). Using GPUs significantly reduces the flops per dollar ratio (Chien-Ping, 2010), thus making intensive neuronal simulations available to a larger audience.

Our application uses NEURON (Carnevale and Hines, 2006) for constructing the equation system from the neuron morphology and solves the system on the GPU. To validate the correctness of our model, we compared our results to those calculated in NEURON, resulting in a small error between the platforms (Figure 2), which decreased with smaller step size. The error probably arises from three factors: (1) CUDA runs in single precision while NEURON runs in double precision. (2) Using the hardware “fast math” optimization (NVIDIA, 2011b) reduces accuracy of math operations. (3) Stone's algorithm (Stone, 1973) uses a large number of multiplications, thus propagating the errors arising from 1 and 2.

Prior to implementing the simulation environment on the GPU we assumed that most of the computation time would be used in solving the current diffusion equation system (or the matrix). This was confirmed by our runtime analysis of the application, this task consuming 54–56% of the runtime (Figure 3). We thus focused on solving the matrix more efficiently. In the theory presented here, we introduced two methods for the back-substitution part of solving the equation system—segment-based (Figure 1) and branching-based parallelization. The branching-based method performed better for highly branched morphologies, while the segment-based method was best for relatively simple morphologies (Figure 4). The branching-based parallelization used less framework shared memory (see Methods) than the segment-based method. This may allow simulation of more sweeps per blocks, which means running heavier simulations faster.

After validating the correctness of our application we explored the domains of applicability, domains in which our simulations performed better than NEURON. We also examined for which cases each back-substitution method should be used. The most basic simulation used a fork morphology and a single sweep (Figure 4A) and reached a speedup of 1.8-fold when using the Hodgkin–Huxley model. However, NEURON was 5.3 times faster using the passive model. This difference in performance was due to the advantages of the GPU with more complex models, since the math operations reduce the memory transfer latency [for more details on memory latency on GPU see Nickolls et al. (2008); Ryoo et al. (2008)].

We next checked more realistic simulations using morphologies reconstructed from real neurons and each simulation composed of several traces. When NEURON simulates multiple traces on the same neuron, the increase in performance time shows a constant relationship to the number of data points. In contrast, in CUDA the increase in runtime was sub-linear (Figure 5A). With 13 traces CUDA ran 14.6-fold faster than NEURON. The application's runtime did not increase linearly with increase in trace/data points, since the simulating multiprocessor was occasionally idle and could context switch between warps while waiting for memory transfers.

The final simulation we ran, used multiple sweeps for many neurons. Here our application reached a peak of 150-fold faster than NEURON. The increase in speed arose from each neuron being simulated on one of the 14 different multiprocessors composing the GPU. Figure 5B shows that runtime increased in multiples of 14 steps, since runtime was set by the multiprocessor that finished last. Simulating many instances of the same neuron expediently, opens the door to apply Monte Carlo simulations for statistical analysis and optimization algorithms—GPUs outmatch most platforms for these kind of problems (Lee et al., 2010; Quinn and Abarbanel, 2011).

Using GPUs for simulating compartmental models may thus decrease runtime to the order of 2 magnitudes. Naive optimization using openCL or running the same algorithm used by the CPU code converted to a GPU may achieve only minor speedups in the order of one magnitude. Converted code from CPU to GPU cannot use the advantages of the SIMD (single instruction multiple data) architecture of the GPU. Our application, however, was tailored to the GPU and implemented several optimizations to take advantage of its computational power. For example, ILP (Volkov, 2010) dealt with multiple segments, so each thread calculated several segments. Using ILP allowed us to use a fixed number of threads (32—a single warp) to simulate the most basic simulation unit—one sweep. Using one warp for the whole sweep fixed CUDA block's x-index to 32, where the y-index was the number of sweeps in the protocol (see Methods). This layout was optimal in our application and spared the user the need to find the optimal number of threads in the block, as in our previous GPU ion cannel simulator (Ben-Shalom et al., 2012).

We gained a further increase in performance by using a buffer shared memory of the size of a warp that held the output of the recorded site in the simulation. This buffer was filled every 32 time steps by the different threads in the warps and then transferred the information to the global memory. This optimization assured that slow global memory transfers occurred only every 32nd step and would be coalesced—uncoalesced memory transfer are major slowdown factors in CUDA applications (Ryoo et al., 2008).

NEURON is a comprehensive solution for simulations in neuroscience. Our application does not replace NEURON. Instead, we suggest the use of GPUs for compartmental modeling. In the future we hope to incorporate our application into NEURON, to allow electrophysiologists to combine the variety of tools NEURON offers with the low budget speed gain of GPUs presented here. Currently users can extend our application for their needs by adding membrane mechanisms using our implementation of the Hodgkin–Huxley model as an example.

A further natural extension would be to simulate realistic neuronal networks on GPUs. This would result in a supercomputer with the computational abilities of the expensive Blue Gene (Gara et al., 2005), allowing laboratories with a much lower budget access to supercomputing. Until neuronal network simulation is possible on GPUs, one can use hybrid CPU–GPU supercomputers where the complicated neurons may be simulated using our application, while the CPUs simulate the network organization and communication between the neurons.

The aim of this study was to construct a software that uses GPUs for simulating compartmental models. GPUs are evolving very quickly. NVIDIA released the new Kepler architecture (NVIDIA, 2012) only 2 years after the Fermi architecture (NVIDIA, 2009). This new architecture is more power efficient and nearly doubles performance—3090 GFLOPS compared to 1581 of the Fermi architecture (NVIDIA, 2012). CUDA's scalability permits easy implementation of our application to the new and future architectures. Easy scalability assures that the application will perform even better with GPU development.

### Conflict of interest statement

The authors declare that the research was conducted in the absence of any commercial or financial relationships that could be construed as a potential conflict of interest.
